# Is sub-national healthcare social protection sufficient for protecting rare disease patients? the case of China

**DOI:** 10.3389/fpubh.2023.1198368

**Published:** 2023-06-16

**Authors:** Juan Xu, Mingren Yu, Zhiguo Zhang, Shiwei Gong, Bingqin Li

**Affiliations:** ^1^School of Medicine and Health Management, Tongji Medical College of Huazhong University of Science and Technology, Wuhan, China; ^2^Hubei Provincial Research Center for Health Technology Assessment, Wuhan, China; ^3^Tongji Hospital Affiliated to Tongji Medical College, Huazhong University of Science and Technology, Wuhan, China; ^4^School of Pharmacy, Tongji Medical College of Huazhong University of Science and Technology, Wuhan, China; ^5^Social Policy Research Centre, University of New South Wales, Sydney, NSW, Australia; China Studies Centre, Sydney University, Sydney, NSW, Australia

**Keywords:** rare disease, orphan drug, healthcare safety net, national basic medical insurance, China

## Abstract

**Background:**

Failing to provide social support to cover healthcare costs for rare diseases would lead to great financial distress for the patients and their families. People from countries without a well-developed health safety-net are particularly vulnerable. Existing literature on rare diseases in China focuses on the unmet needs for care of the patients and the difficulties of caregivers and physicians. Very few studies examine the state of social safety-net, the unresolved issues and whether the current localized arrangements are sufficient. This study aimed to gain in-depth knowledge of the current policy system and make sense of the local varieties, which would be essential for developing strategies for future policy changes.

**Methods:**

This systematic policy review focuses on the provincial level policies on subsidizing the healthcare costs for people with rare diseases in China. The cut-off point for the policies was March 19, 2022. The researchers coded the healthcare cost reimbursement policies and identified the different provincial level models based on the usage of reimbursement components in each provinces reimbursement arrangements.

**Results:**

257 documents were collected. Five provincial level models (Process I, II, III, IV and V) have been identified with the five components across the country: Basic Medical Insurance for Outpatient Special Diseases (OSD), Catastrophic Medical Insurance for Rare Diseases (CMIRD), Medical Assistance for Rare Diseases (MARD), Special Fund for Rare Diseases (SFRD) and Mutual Medical Fund (MMF). The local health safety-net in each region is a combination of one or more of the five processes. Regions vary greatly in their rare diseases coverage and reimbursement policies.

**Conclusion:**

In China, the provincial health authorities have developed some level of social protection for rare disease patients. However, there are still gaps regarding coverage and regional inequality; and there is room for a more integrated healthcare safety-net for people suffering from rare diseases at the national level.

## 1. Introduction

Rare diseases have low morbidity but severe clinical manifestations, with most cases being genetic and often pediatric onset (1, 2). It is estimated that between 260 and 450 million people suffer from 5,000–8,000 rare diseases worldwide (2, 3). Thanks to the media exposure, there is growing public awareness of some rare diseases (such as phenylketonuria, Gaucher disease, etc.) (4). However, insufficient knowledge on etiology and pathogenesis often results in misdiagnosis, missed diagnosis, or incurability (5, 6). The patients and their families have to endure the physical and financial burdens of the diseases and often find themselves to be socially isolated (1, 7).

Orphan drugs and special therapies are essential for the treatment and maintenance of rare disease patients. However, only 5% of rare diseases has approved pharmacotherapies and the orphan drugs are often unaffordable (8). According to EvaluatePharma, an organization specialized in in evaluating and research on drugs and produce market forecasts about drugs, the average spending on orphan drugs per patient for the top 100 US orphan drugs was almost 4.5 times higher than the non-orphan drugs in 2018, and the sales of orphan drugs are expected to reach USD 242 billion, capturing one-fifth of worldwide prescription sales by 2024 (9). The high costs would result in severe financial burdens for families with rare disease patients. Therefore, in many parts of the world, there have been government policies and medical insurance schemes to fully cover or subsidise part of the costs of treatment or therapies (4, 10, 11).

In developing countries where there is not yet fully developed healthcare and welfare systems, a full-fledged social protection system for people with rare diseases may not exist. As argued by Groft, et al. (12), “There is a need to expand awareness, advocacy, and outreach to everyone including those with low incomes, poor literacy, minority ethnic status and living in underserved and marginalised populations in urban and rural areas as well as in developing nations throughout the world.” It is also important to note that as poor countries develop economically and the public awareness of rare diseases improves, the social need and political pressure for providing healthcare and maintenance coverage for rare disease patients are also on the rise. Without a sustainable financial arrangement for countries and for families, both the health service providers and service users will be caught in great social tensions. To improve the situations, policy makers indeed have tried to make some efforts. It is important to examine how countries deal with the challenges and what principles they use (13). As each country can be different, it is worthwhile to conduct detailed case studies to gain deeper understanding of the practices, the principles and remaining debates.

This paper focuses on the practices of China. An estimate, using the rather conservative thresholds of affecting less than 1 in 500,000 people or less than 1 in 10,000 newborns (14), showed that at least 16.8 million patients in China suffered from one or more rare diseases (~1.2 percent of the Chinese population living with rare diseases). Whereas, in developed countries, the definition is stricter, for example, EU define rare diseases as affecting less than 1 in 2000 people (15). The people living with rare diseases can be as about 8 percent of the total population in EU (2).

A 2018 survey in China found that the average out-of-pocket costs (OOP) paid by patients with rare diseases exceeded CNY 40,000 annually (5). The data from the National Bureau of Statistics in China shows that in the same year, the *per capita* household consumption was CNY 19,853 and the average household consumption should be CNY 79, 412, assuming four person per household (16). This means that for an average family, if one member suffers from a rare disease, the healthcare costs for the patient would be equivalent to nearly half of the household consumption. According to the definition of World Health Organization, when household medical expenditures exceed 40% of household consumption, it would be a case of catastrophic health expenditure (17). The costs for some lifesaving drugs, i.e., the orphan drugs, can be very costly. For example, the annual costs for imiglucerase (Cerezyme^®^) and for Gaucher disease can be as expensive as CNY 2.65 million, according to the latest winning bids to supply imiglucerase in China (18, 19). This means even a high-income family can find the costs unbearable.

To solve the problem, the Chinese government started to introduce medical insurance coverage. The basic medical insurance system in China has two sub-schemes: the Urban Employee Basic Medical Insurance (UEBMI) and the Urban and Rural Residents Basic Medical Insurance (URRBMI) (20, 21). An official National Reimbursement Drug List (NRDL) defines what drugs can receive insurance coverage. The NRDL concentrates on common diseases and frequently occurring conditions. However, the NRDL included drugs that can be used to treat only 29 rare diseases (22). A Catastrophic Medical Insurance (CMI) then was introduced at the provincial level in 2012 and at the national level in 2016 to reduce medical costs for patients with serious diseases (23). Even with the CMI, in 2018, the national healthcare safety-net only covers 20% of the total treatment costs (TTC) for rare disease patients (5). As the national level reimbursement is very low, provincial authorities had to set up local healthcare safety-nets (4, 21). They adapted the local medical care social insurance schemes to reimburse the extra amount that the patients have to bear after the national medical social insurance coverage. However, since the provincial authorities have to face regional constraints, such as socio-demographic factors, public opinions and local fiscal capacity, local governments do not offer the same coverage.

Some existing studies discussed the healthcare safety-nets for rare diseases in China, but only several provinces have been analyzed (24–27). There is no systematic comparison of provincial level practices nationwide. This study fills in the research gap and presents a detailed mapping of the healthcare safety-nets for rare diseases in 31 provincial level jurisdictions in China. Apart from providing a structured overview of the system, we also comment on the strengths and gaps in the current system. This research contributes to the emerging research on the social projection of rare diseases in developing context. It does not only provide information on the different local models, but also highlights the embedded issues of central-local division of responsibilities in developing social insurance funding pools.

## 2. Methods

The policies reviewed in this article are published by the provincial-level People’s Governments, Healthcare Security Administrations, Human Resources and Social Security Departments and Civil Affairs Breaux of 31 provinces (autonomous regions, municipalities). We only included these regions because they share the same healthcare system, which would allow us to compare regional variations. The review’s cutoff point was March 19, 2022.

Since the definition of “rare disease” is inconsistent globally (28), we use the definition in the *First List of Rare Diseases of China (Zhongguo Diyipi Hanjianbing Mulu)* to conduct the policy searching. The implication is that rare health conditions reimbursed by some local policies but do not appear in *the First List of Rare Diseases of China* are not included in this study. However, as the paper intends to identify the principles and models of coverage, including more diseases in the analyses would not add much more information. 257 documents were collected and, the content of these policies was coded according to the following themes:

(a) The components of the healthcare safety net for rare diseases;
(b) The number of rare diseases covered in the healthcare safety net;
(c) The reimbursement policy for the health service costs and orphan drugs; and,
(d) The inclusion criteria for determining rare diseases and the orphan drugs reimbursed.

## 3. Results

**Figure 1:**
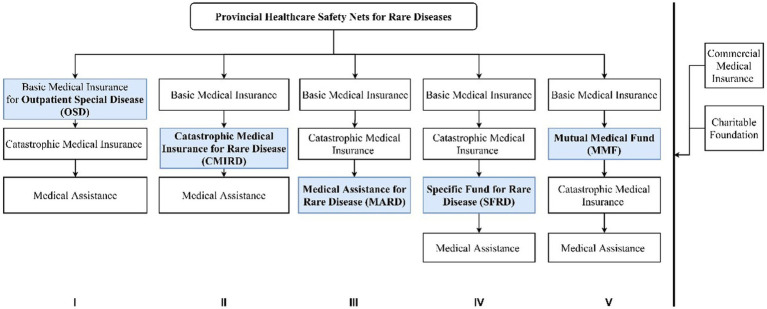
Reimbursement components and processes to cover rare diseases. Source: Compiled by the authors.

Although there is no dedicated overarching healthcare safety net for rare diseases, provincial level authorities have made efforts to explore solutions to address healthcare affordability issues. In a nutshell, they combine several types of reimbursement components into reimbursement processes, which allow them to use funding from multiple sources. Figure 1 summarizes the relationship between the five reimbursement components and the five processes.

### 3.1. Reimbursement components

#### 3.1.1. Basic medical insurance

All provinces have Basic Medical insurance. Basic medical insurance for Outpatient Special Diseases (OSD) can cover chronic and severe diseases that may incur a heavy economic burden and can be treated with drug therapies in the outpatient settings. The use of OSD is only limited to medicines listed in the NRDL.

Local governments make adaptations to OSD to allow it to offer more support to patients with rare diseases. The first is resetting the deductible rate, the co-payment rate, and the highest amount of reimbursement for rare disease patients. For example, the annual maximum amount of the reimbursement for a general outpatient in Wuhan City, Hubei Province, was CNY 400 in 2020. The maximum is higher for some rare diseases, e.g., CNY 5,000 for Parkinson’s disease or CNY 20,000 for hemophilia. Lowering the deductible rate, raising the co-payment rate, and the maximum amount of reimbursement can lower the costs of healthcare costs for patients with rare diseases. The second is reclassifying the outpatient expenditures of rare disease patients as inpatient expenditures so that the patients can be reimbursed by basic medical insurance and catastrophic medical insurance with a much higher co-payment ratio and the maximum amount for the reimbursement. For example, in Beijing, the outpatients with Hemophilia are entitled to be reimbursed at the rate of inpatients at nearly 80 percent and with the maximum amount of CNY 250,000. The third is introducing a separate system for orphan drugs only inside the basic medical insurance. In this sub-scheme, costs for orphan drugs would be reimbursed separately from the healthcare service expenditures, usually with a better term of reimbursement. For example, in the OSD of Fujian province, patients with Niemann-Pick disease could access special reimbursement for Miglustat. The special reimbursement scheme enjoys no deductible amount, a higher co-payment ratio at 80% and an annual reimbursement cap higher than common disease patients. This sub-scheme of OSD is called Special Medicine Management in some provinces, like Fujian, Sichuan, Xinjiang and other provinces.

#### 3.1.2. Catastrophic medical insurance

Catastrophic Medical Insurance is designed to reduce the catastrophic disease burdens. Some provinces do not differentiate catastrophic medical insurances for common diseases and for rare diseases (CMIRD). But some do differentiate the two. With this component, a rare disease patient may receive substantial financial support.

The rare diseases that need orphan drugs are covered by the CMIRD, depending on the successful negotiation between the provincial Healthcare Security Administration and the pharmaceutical companies. The negotiation aims to cover the expensive orphan drugs that are usually excluded from the NRDL. If the negotiation is successful, the price of orphan drugs can be reduced sharply, and the financial burden of rare diseases will also decrease.

The rare diseases and related orphan drugs are usually listed separately in the CMIRD with a much higher co-payment ratio and cap of the reimbursement amount, compared with the common diseases covered in the CMI. For example, in Shandong province, the highest amount of reimbursement for rare diseases (e.g., Gaucher disease and its specific orphan drug, imiglucerase) in the special CMIRD is CNY 900, 000, whereas the amount is CNY 400, 000 for standard catastrophic medical insurance.

*Medical Assistance*. Medical assistance is an important part of the national health security system for the insured. Only several provinces, including Hunan and Guangdong, have established medical assistance for rare diseases (MARD) specifically. After being co-paid by the basic medical insurance and CMI, the unpaid medical costs of patients with rare diseases can be compensated by MARD. Although the reimbursement of MARD is capped at a fixed amount, coverage is not limited to the medicines in the National List, and it provides patients of rare diseases with significant additional help to reduce their financial burden. MARD in some provinces also allows a further deduction of the treatment costs in the process of the basic medical insurance and catastrophic medical insurance. The special reimbursement policy usually includes removing the deductible cost and increasing the co-payment ratio and the maximum reimbursable amount.

#### 3.1.3. Special fund for rare diseases (SFRD)

The funding of SFRD comes from the government’s fiscal special fund or the fund pool of CMI, which is why some people think it is the same as CMIRD. However, we must give this component special attention for its potential to evolve into a dedicated healthcare safety net for rare diseases in China. Up to now, Shanxi, Zhejiang and Jiangsu provinces have established a SFRD, and Sichuan province is in the process of introducing it. The benefit of a SFRD is that the ringfenced funding eliminated the financial competition between rare diseases and common diseases. For example, in Zhejiang province, part of the CMI will be reserved for SFRD, with the amount of CNY 2 per insured person every year. Only several very expensive orphan drugs that are not included in the NRDL can be reimbursed with SFRD. The separately fund pool also means SFRD can compensate the drug costs at a much higher level. For example, in Zhejiang province, thanks to the SFRD, patients suffering from Gaucher disease who used to face a hefty charge of CNY 2.65 million each year for imiglucerase (Cerezyme®) need only to pay for a maximum of CNY 100,000 every year.

#### 3.1.4. Mutual nedical fund (MMF)

The funding of MMF is mainly raised from parents, and it covers all infants, children, and school-age students, regardless of their health status. The MMF is not directed at rare diseases, but it does play a crucial supplementary role in the healthcare safety net for rare diseases. MMF is currently established in Shanghai only, under the control of the Red Cross of China Shanghai Branch. Since most rare diseases are inherited, the Shanghai government considers that helping newborns and children with rare diseases from poorer backgrounds with inadequate health resources is a priority. What is more, the government intends to integrate the resources and power of society, particularly families, in its creation of a specific additional health security system for newborns and children with rare diseases. The cap of the reimbursement amount of MMF is rather small compared to the other components, but the diseases covered are not limited to those in the NRDL.

In total, 30 provinces (96.8%) included OSD in their special healthcare safety net for rare diseases, 5 provinces (16.1%) with CMIRD, 9 provinces (29.0%) with MARD, and 4 provinces (12.9%) with SFRD. Only Shanghai introduced MMF (3.2%).

### 3.2. Reimbursement processes

Reimbursement processes show the relationship between different reimbursement components, including which components and the sequence of using the components. The policy review shows that the 31 provincial regions picked and mixed the five components to form five distinctive processes of reimbursement. As shown in Figure 1, Processes I, II and III share similar components of basic medical insurance, catastrophic medical insurance, and medical assistance. What makes them different from each other is where the ringfenced pool of funds for rare diseases is located. In Process I, the ringfenced rare disease funding pool is inside the basic medical insurance; in Process II, the pool is in catastrophic medical insurance; and in Process III, the pool is in medical assistance. As discussed earlier, basic medical insurance provides protection for a set of 29 rare diseases and the financial support is rather insufficient. In contrast, catastrophic medical insurance provides more coverage for expensive services and drugs in its own list. Medical assistance does not offer a large amount of support but there is no constraint of a medicine list. Process IV and Process V do not embed the rare disease in the existing system, the ringfenced funding pool sits completely outside the state social insurance schemes. The special fund in Process IV came from local budgets and the mutual medical fund in Process V came from parental contributions.

Therefore, the five processes represent not only the different amount of coverage, but the principles are also somewhat different. Social insurances and mutual funds stress personal responsibilities and mutual support. Assistance and special funds are public aids to the neediest. Therefore, it is not difficult to tell why assistance and special funds came after social insurances in all five processes, as the overall attitudes of the local governments is to encourage personal contribution as much as possible.

In terms of level of reimbursement, Processes I, II, and IV have provided the highest level of protection. Process IV allows the out-of-pocket expenses for a patient to be capped at the maximum of CNY 100,000 each year, irrespective of the total costs. A province may have several reimbursement processes running parallel to each other. For example, Zhejiang Province uses I, III, and IV. 35.5 percent of the provinces have at least two reimbursement processes, and 64.5 percent uses Process I only. The special healthcare safety net for rare diseases and the processes involved is shown in detail in Appendix 1.

### 3.3. Types of rare diseases covered

**Figure 2:**
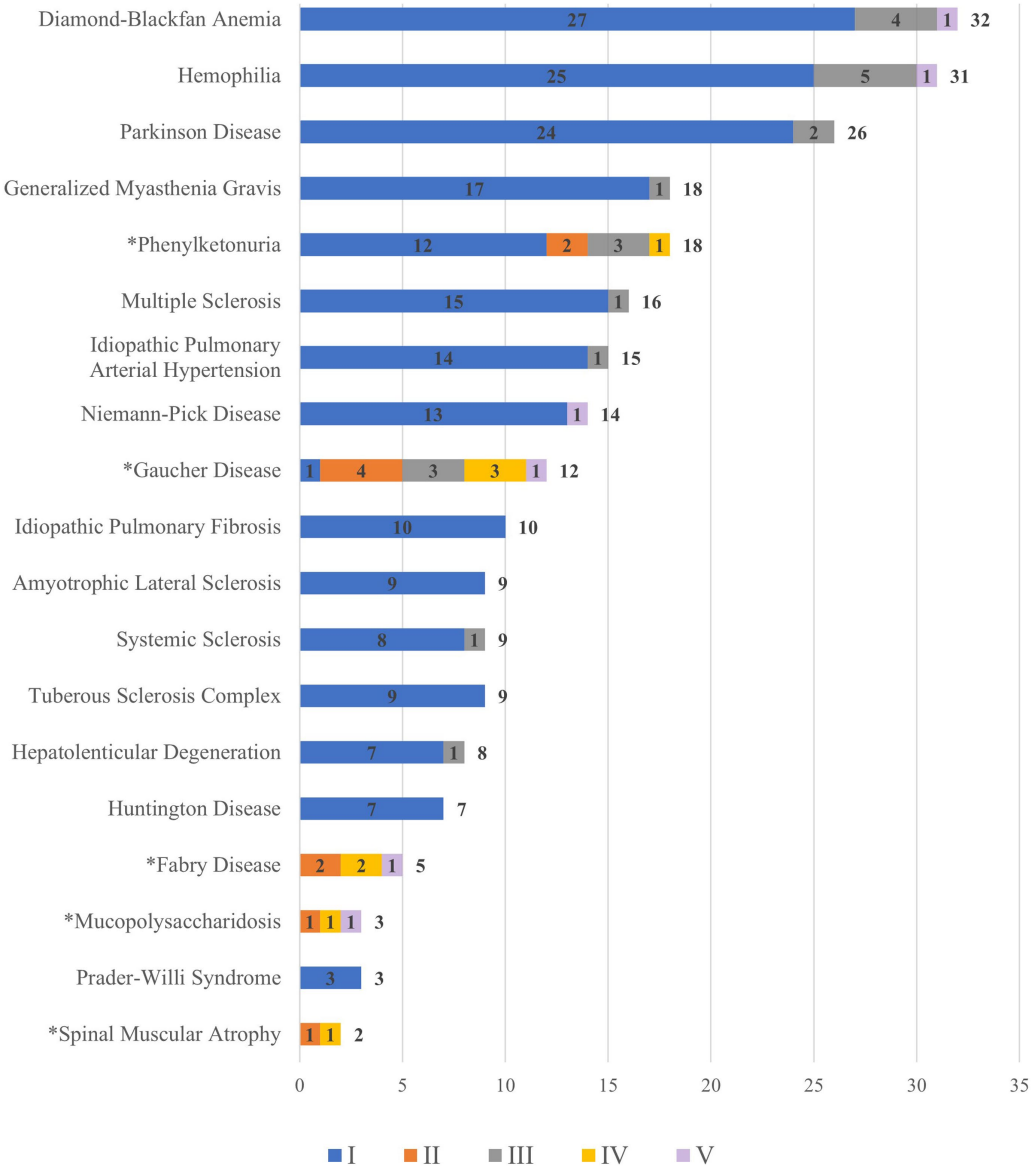
Rare diseases covered by processes and number of provinces. ^a^ For rare diseases with an asterisk (^*^), orphan drugs are excluded from the NRDL. ^b^ The colours of each bar indicates the different elements of reimbursement. The numbers in the middle of the colored bars are the frequency of a rare disease mentioned independently in a reimbursement process in all provinces. ^c^ One rare disease can be mentioned in multiple processes so that the total can be larger than 31, the total number of regions. For example, Diamond-Blackfan Anemia can be counted multiple times because it is covered by different reimbursement processes (I and II) in some province. Therefore, the total frequency of Diamond-Blackfan Anemia is 32. ^d^ We only counted instances when a rare disease is singled out in a policy, not when it is discussed in generic terms referring to a group of diseases. ^e^ The total count of a disease specifically is placed at the right hand of the bars in the chart. Data source: compiled and coded by the authors using provincial level policies (please refer to details in the methodology section).

As the provincial level governments shoulder a large part of the financial responsibilities, they exercise discretions when deciding what diseases to cover. Figure 2 shows the rare diseases covered in each province’s policies and counted how many provinces cover each type of disease.

As shown in Figure 2, Diamond-Blackfan Anemia, Hemophilia (Human Coagulation Factor VII and IX, Recombinant Human Coagulation Factor VIIa for Injection), Parkinson Disease (Levodopa), Generalized Myasthenia Gravis (Pyridostigmine Bromide Tablets) and Phenylketonuria (Sapropterin) are covered by more provinces than other diseases. The rare diseases whose specific orphan drugs are not covered in the NRDL are indicated with an asterisk (*). Therefore, the patients who would suffer from unaffordable drugs are those with phenylketonuria (Sapropterin), Gaucher disease (imiglucerase), and Fabry disease (agalsidase beta and agalsidase alpha).

### 3.4. Regional variations in reimbursement policies

Provincial level health authorities exercise discrepancy regarding reimbursement policies on the deductible amount, co-payment ratio, and maximum amount. Each reimbursement process has its own policies to cover healthcare and orphan drugs. The level of coverage can be different by province (Table 1). Healthcare services and orphan drugs can be treated differently using different reimbursement processes even if they are in the same region.

**Table 1:** The comparison of reimbursement policies between different special healthcare safety nets for rare diseases.

Reimbursement processes	Example provinces	Example rare diseases	Specific orphan drug^a^	Characteristics of the reimbursement processes	Deductible (CNY)	Co-payment ratio (costs = X, unit: CNY)	Reimbursement Cap (CNY)
I	Beijing	Hemophilia	Y	Outpatient expenditures reimbursed as inpatient expenditure	1,300	78% of X (residents); 85% ~ 95% of X (employees)	250,000 (residents); 500,000 (employees)
	Hubei	Hemophilia	Y	Outpatient expenditures reimbursed with a higher reimbursement amount	Not set	50% of X (residents); 60% of X(employees)	16,000 (residents); 20,000 for employees
	Fujian	Niemann-Pick disease	Y	Reimbursing medicine costs separately	Not set	80% of X	same as an insured^b^
II	Shanxi	Gaucher Disease	N	Reimbursing medicine costs through catastrophic medical insurance	Not set	50% of X	400,000
	Shandong	Gaucher Disease	N	Reimbursing medicine costs through catastrophic medical insurance	20,000	80% if 20,000 < X ≤ 400,000; 85% if X > 400,000	900,000
III	Beijing	Hemophilia	Y	Reimbursing medical costs after being reimbursed by other medical insurance	Not set	75% of X	80,000
	Qinghai	Hemophilia	Y	Reimbursing medical costs after being reimbursed by other medical insurance	Not set	90% of X	10,000
IV	Shanghai	Gaucher Disease	N	Reimbursing medicine costs after being reimbursed by basic medical insurance	300	50% of X	100,000
V	Zhejiang	Gaucher Disease	N	Reimbursing medicine costs through new funds separated from catastrophic medical insurance	NA	80% if X ≤ 300,000; 90% if 300,000 < X ≤ 700,000; 100% if X > 700,000	NA
	Shanxi	Gaucher Disease	N	Reimbursing medicine costs through a special fiscal fund	NA	60% of X	NA

a Y: Specific orphan drugs are covered in the NRDL; N: Specific orphan drugs are not included in the NRDL.

b The orphan drug expenditure shares the same capped amount of reimbursement with other medicines cost.

### 3.5. Inclusion criteria

Sixteen provinces publicized the criteria for including a rare disease. The provinces include Shanghai, Chongqing, Shanxi, Shaanxi, Shandong, Fujian, Zhejiang, Henan, Hunan, Anhui, Hainan, Sichuan, Guizhou, Yunnan, Inner Mongolia, and Xinjiang. The policies in these provinces mentioned seven criteria:

(a) There exist special medicines for the disease, and their clinical efficacy is proven (11);
(b) The economic burden of the disease is substantial (10);
(d) The disease is severe, or the course of the disease is long (8);
(d) Suitable outpatient services can be provided for treating the disease (8);
(e) The specific drugs are covered in the NRDL (7);
(f) The financial risks of including orphan drugs in the health insurance is manageable (5);
(g) There is high media exposure and good public awareness of the disease (3).

The numbers in brackets indicate how many provinces have adopted this criterion. Clinical efficacy and economic burden of rare diseases are the leading concerns.

## 4. Discussion

Rare diseases are costly to treat and manage. Failing to provide social protection for patients would result in families being trapped in financial difficulties or patients left untreated. This article reviews the emerging provincial level healthcare safety nets for rare diseases in China and for the first time, all provinces in mainland China are studied using one framework of analysis. It generates important insights on the status quo of the system and identifies local variations in the principles and practices in protecting patients and families against the financial shock caused by rare diseases.

This research establishes that a local rare disease health safety net gradually emerges in China. However, China has not yet legislated to control the costs of orphan drugs and not initiated a national level rare disease protection system (11, 29, 30). The absence of national guideline limits the development of regional medical systems for rare diseases and leave reimbursement processes unchanged (6, 24). For example, the SFRD process was found nonexistent in 27 provinces. In a few wealthy provinces in Zhejiang and Jiangsu, SFRD was only established w in 2019 and 2021, respectively. Different from other policy fields in which the central government set up a policy framework and local governments operationalize (31), the healthcare safety-net for rare diseases was developed locally without an overarching framework at the Center. This resulted in regional variations.

The significant result of this policy review is to identify the pattern for local variations. Five reimbursement components OSD, CMIRD, MARD, SFRD, MMF can be picked and mixed into five Reimbursement Processes I, II, III, IV and V. A common feature for all regions is that they all adopted a hybrid solution to the problems of the high healthcare costs and the super expensive orphan drugs. In each region, the solution may include basic medical insurance, catastrophic medical insurance, medical assistance and so on. The hybrid system focuses on rare diseases, provides a targeted and higher-level protection and introduces caps for out-of-pocket expenditures. Each province has their disease list and orphan drug list. The drugs covered in the provincial level systems are not limited to the drugs covered by the National Basic Medical Insurance. Drugs outside the NRDL may also be included. The benefit of such a hybrid and localized system is that it is more flexible and allows local governments to adjust policies according to local circumstances. This approach is particularly useful when there are regional variations in the types and burdens of rare diseases. However, the system is highly unequal. In most regions, a patient could at best rely on OSD to get a couple hundred thousand yuan covered. A national-level arrangement that would allow redistribution between wealthier and poorer regions may address inequality. However, this would require national-level policymaking, concentrating on the reimbursement processes and policies, rare diseases covered and inclusion criteria. Another justification for an overarching policy framework at the national level is that it would also help to overcome confusion as to who should cover what at different levels.

It is important to note certain issues regarding social protection for rare diseases and acknowledge the limitations of this study. Firstly, this research does not consider private medical insurance, which can serve as a supplementary to social protection. Given the availability of various private medical insurances across the 31 provinces/regions, conducting affordability analyses based on both public and private insurance coverages would be necessary to determine the national-level framework required and define central-local responsibilities more clearly. Secondly, this research is on social protection against very high healthcare and drug costs. Using social protection systems to cover these costs is only the demand side solution which means the public and social funds are used to improve affordability. This line of thinking does not address the supply side issues. As pointed out in the existing literature, there is limited effort from the governments to improve accessibility, i.e., providing better information on treatment and drugs (32). There also needs to be greater efforts by the government to address the affordability issue by tackling drug pricing directly. This can be done through more diligent price negotiation and controlling the drug sellers’ profit margin as done in many other countries in the world. Tackling the problems on both sides can avoid the inefficiency of spending large quantities of the very limited social health funds on overpriced drugs and services.

## 5. Conclusion

In China, the provincial health authorities have developed some level of social protection for rare disease patients. However, there are still gaps regarding coverage and regional inequality and there is room for a more integrated healthcare safety-net for people suffering from rare diseases at the national level.

## Author contributions

JX and MY led the conceptualization, research, and writing of the article. JX, MY, ZZ, SG, and BL contributed to manuscript writing and revisions. All authors contributed to the article and approved the submitted version.

## Conflict of interest

The authors declare that the research was conducted in the absence of any commercial or financial relationships that could be construed as a potential conflict of interest.

## Publisher’s note

All claims expressed in this article are solely those of the authors and do not necessarily represent those of their affiliated organizations, or those of the publisher, the editors and the reviewers. Any product that may be evaluated in this article, or claim that may be made by its manufacturer, is not guaranteed or endorsed by the publisher.

## Supplementary material

The Supplementary material for this article can be found online at: https://www.frontiersin.org/articles/10.3389/fpubh.2023.1198368/full#supplementary-material

## Abbreviations

CMI: Catastrophic medical insurance
CMIRD: Catastrophic medical insurance for rare diseases
MARD: Medical assistance for rare diseases
MMF: Mutual medical fund
NRDL: National reimbursement drug list
OOP: Out-of-pocket costs
OSD: Outpatient special diseases
SFRD: Special fund for rare diseases
TTC: Total treatment costs
UEBMI: Urban employee basic medical insurance
URRBMI: Urban and rural residents basic medical insurance
