# Supplementary material for: Is sub-national healthcare social protection sufficient for protecting rare disease patients? the case of China
Source: Front Public Health. 2023 Jun 16;11:1198368. doi: 10.3389/fpubh.2023.1198368 (PMC10311551; doi:10.3389/fpubh.2023.1198368)
Supplement: Supplementary file 1 [file Table_1.DOCX]

Supplementary Material

**Is Sub-national Healthcare Social Protection Sufficient for Protecting Rare Disease Patients? The case of China**

Juan Xu†, Mingren Yu†, Zhiguo Zhang, Shiwei Gong, Bingqin Li*

*** Correspondence:** Bingqin Li [Bingqin.li@unsw.edu.au](mailto:Bingqin.li@unsw.edu.au)

**Appendix 1 The healthcare safety net for rare diseases in different provinces**

| **Provinces** | **The reimbursement processes** | **The rare diseases covered** |
| --- | --- | --- |
| Beijing | (a)I | (a)DBA, Hemo, IPAH, IPF, MS, NPD; |
|  | (c)III | (c)DBA, Hemo |
| Tianjin | (a)I | (a)DBA, Hemo |
| Shanghai* | (e)V | (e)DBA, FD, GD, Hemo, MPS, NPD |
| Chongqing | (a)I | (a)ALS, DBA, GMG, Hemo, HLD, PD, PKU, PWS |
| Hebei | (a)I | (a)DBA, GMG, Hemo, IPAH, MS, SS |
| Shanxi | (a)I | (a)ALS, DBA, HD, Hemo, IPAH, IPF, MS, NPD, TSC; |
|  | (b)II | (b)GD; |
|  | (c)III | (d)GD; |
|  | (d)IV | (e)GD |
| Jilin | (a)I | (a)DBA, GMG, Hemo, HLD, IPF, MS, PKU, PD, TSC; |
| Liaoning | (a)I | (a)DBA, GMG, Hemo, PD; |
|  | (b)II | (b)PKU |
| Heilongjiang | (a)I | (a)DBA, PD |
| Shaanxi | (a)I | (a)DBA, Hemo, PD, PKU; |
|  | (b)II | (b)FD, GD, MPS, SMA |
| Gansu | (a)I | (a)DBA, GMG, Hemo, PKU, PD |
| Qinghai | (a)I | (a)DBA, Hemo, PD; |
|  | (c)III | (c)DBA, Hemo, PD |
| Shandong | (a)I | (a)DBA, GMG, Hemo, HLD, PD, PKU; |
|  | (b)II | (b)FD, GD, PKU; |
|  | (c)III | (c)PKU |
| Fujian | (a)I | (a)ALS, DBA, GMG, HD, Hemo, IPAH, PD, PKU, NPD, MS, SS, TSC |
| Zhejiang | (a)I; (c)III** | (a)PD; |
|  | (d)IV | (d)FD, GD, PKU |
| Henan | (a)I | (a)ALS, DBA, Hemo, IPAH, IPF, MS, NPD, PD, PKU, TSC |
| Hubei | (a)I | (a)DBA, Hemo, PD, PKU |
| Hunan | (a)I | (a)DBA, GD, GMG, Hemo, HLD, IPAH, MS, NPD, PD, PKU, SS, TSC; |
|  | (b)II | (b)GD; |
|  | (c)III | (c)DBA, GD, GMG, Hemo, HLD, IPAH, MS, PD, PKU, SS |
| Jiangxi | (a)I | (a)DBA, GMG, Hemo, PD |
| Jiangsu | (a)I | (a)ALS, HD, Hemo, IPAH, MS, NPD, PKU, TSC; |
|  | (c)III | (c)DBA, Hemo; |
|  | (d)IV | (d)FD, GD, MPS, SMA |
| Anhui | (a)I | (a)ALS, DBA, GMG, Hemo, HLD, IPAH, IPF, MS, NPD, PD, PWS, SS |
| Guangdong | (a)I | (a)DBA, Hemo, IPAH, MS, NPD, PD; |
|  | (c)III | (c)GD |
| Hainan | (a)I | (a)ALS, DBA, GMG, HD, Hemo, IPAH, IPF, MS, NPD, PD, SS |
| Sichuan | (a)I; (d)IV** | (a)ALS, DBA, Hemo, HD, HLD, IPAH, IPF, MS, NPD, PD, PWS, SS, TSC |
| Guizhou | (a)I | (a)DBA, GMG, Hemo, HLD, IPAH, IPF, MS, NPD, PD, PKU, TSC |
| Yunnan | (a)I | (a)DBA, GMG, Hemo, PD, SS; |
| Inner Mongol*** | (a)I | (a)DBA, GMG, HD, IPAH, IPF, MS, NPD, PD, SS, TSC |
| XinJiang | (a)I | (a)ALS, GMG, HD, Hemo, IPAH, IPF, MS, NPD, PD |
| Ningxia | (a)I | (a)DBA, GMG, PD, PKU |
| Guangxi | (a)I | (a)DBA, GMG, Hemo, PD; |
|  | (c)III | (c)Hemo, PKU |
| Tibet*** | (a)I | (a)DBA |

ALS: Amyotrophic Lateral Sclerosis; DBA: Diamond-Blackfan Anemia; FD: Fabry Disease; GD: Gaucher Disease; GMG: Generalized Myasthenia Gravis; Hemo: Hemophilia; HLD: Hepatolenticular Degeneration; HD: Huntington Disease; IPAH: Idiopathic Pulmonary Arterial Hypertension; IPF: Idiopathic Pulmonary Fibrosis; MS: Multiple Sclerosis; MPS: Mucopolysaccharidosis; NPD: Niemann-Pick Disease; PD: Parkinson Disease; PKU: Phenylketonuria; PWS: Prader-Willi Syndrome; SMA: Spinal Muscular Atrophy; SS: Systemic Sclerosis; TSC: Tuberous Sclerosis Complex.

*Shanghai also established I, but no rare disease is covered in the Process I.

**This reimbursement process of the healthcare safety nets for rare diseases in this region is under construction.

***The policy related to the healthcare safety nets for rare diseases cannot be found at the provincial level; related policies were instead collected in the provincial capital city.

**
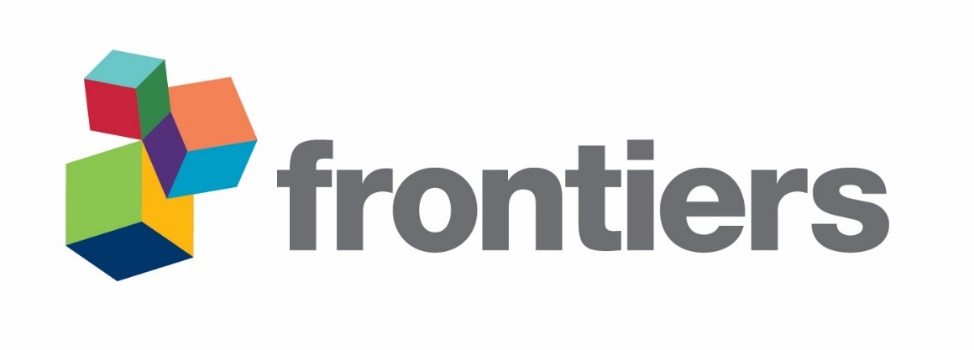
**
